# The Canonical Long-Chain Fatty Acid Sensing Machinery Processes Arachidonic Acid To Inhibit Virulence in Enterohemorrhagic Escherichia coli

**DOI:** 10.1128/mBio.03247-20

**Published:** 2021-01-19

**Authors:** Melissa Ellermann, Angel G. Jimenez, Reed Pifer, Nestor Ruiz, Vanessa Sperandio

**Affiliations:** a Department of Microbiology, University of Texas Southwestern Medical Center, Dallas, Texas, USA; b Department of Biochemistry, University of Texas Southwestern Medical Center, Dallas, Texas, USA; Vanderbilt University; University of Hawaii at Manoa

**Keywords:** infection, host-pathogen interactions, virulence regulation, fatty acid, omega 6, PUFA, arachidonic acid, FadR, enterohemorrhagic *E. coli* (EHEC), locus of enterocyte effacement (LEE)

## Abstract

The mammalian gastrointestinal tract is a complex biochemical organ that generates a diverse milieu of host- and microbe-derived metabolites. In this environment, bacterial pathogens sense and respond to specific stimuli, which are integrated into the regulation of their virulence programs. Previously, we identified the transcription factor FadR, a long-chain fatty acid (LCFA) acyl coenzyme A (acyl-CoA) sensor, as a novel virulence regulator in the human foodborne pathogen enterohemorrhagic Escherichia coli (EHEC). Here, we demonstrate that exogenous LCFAs directly inhibit the locus of enterocyte effacement (LEE) pathogenicity island in EHEC through sensing by FadR. Moreover, in addition to LCFAs that are 18 carbons in length or shorter, we introduce host-derived arachidonic acid (C_20:4_) as an additional LCFA that is recognized by the FadR system in EHEC. We show that arachidonic acid is processed by the acyl-CoA synthetase FadD, which permits binding to FadR and decreases FadR affinity for its target DNA sequences. This interaction enables the transcriptional regulation of FadR-responsive operons by arachidonic acid in EHEC, including the LEE. Finally, we show that arachidonic acid inhibits hallmarks of EHEC disease in a FadR-dependent manner, including EHEC attachment to epithelial cells and the formation of attaching and effacing lesions. Together, our findings delineate a molecular mechanism demonstrating how LCFAs can directly inhibit the virulence of an enteric bacterial pathogen. More broadly, our findings expand the repertoire of ligands sensed by the canonical LFCA sensing machinery in EHEC to include arachidonic acid, an important bioactive lipid that is ubiquitous within host environments.

## Abstract (precis)

Polyunsaturated fatty acids (PUFAs) play important roles in host immunity. Manipulation of lipid content in host tissues through diet or pharmacological interventions is associated with altered severity of various inflammatory diseases.

## INTRODUCTION

The mammalian gastrointestinal (GI) tract is a complex biochemical organ that is home to an endogenous community of microbes known as the gut microbiota and a diverse milieu of small molecules and metabolites derived from host, microbial, and exogenous sources. Upon encountering this stimulus-rich environment, bacterial pathogens sense specific signals through a variety of molecular mechanisms. This often includes the direct recognition of signals by transcription factors in the pathogen, which results in the modulation of expression of target virulence genes. Through these sensing mechanisms, intestinal pathogens integrate the biochemical information of their local environment into the regulation of virulence-associated functions essential for establishing successful infection (1, 2).

Enterohemorrhagic Escherichia coli (EHEC) is an intestinal pathogen that frequently contaminates food sources and causes diarrheal disease in humans (3). Upon ingestion, EHEC infects the large intestines by attaching to colonic epithelial cells and deploying its arsenal of virulence-associated functions in order to rapidly replicate and establish infection. This includes the activation of a type 3 secretion system (T3SS), a molecular syringe and needle-like machine that is encoded within the locus of enterocyte effacement (LEE) pathogenicity island (3). EHEC utilizes the LEE-encoded T3SS to promote its physical attachment to the epithelium by modifying the epithelial cytoskeleton and injecting its cognate receptor Tir to intimately attach to the epithelial membrane and form pedestal-like structures (4). This attachment process effaces the microvilli and generates actin-rich pedestal-like structures underneath the bacterium, which results in the characteristic attaching and effacing (A/E) lesions of EHEC disease (3, 5). The majority of genes carried within the LEE—including genes encoding T3SS structural proteins, translocon proteins that dock the T3SS onto target cells, and effector proteins that are injected into target cells—are essential for pedestal formation and successful enteric infection (3, 6).

**FIG 1:**
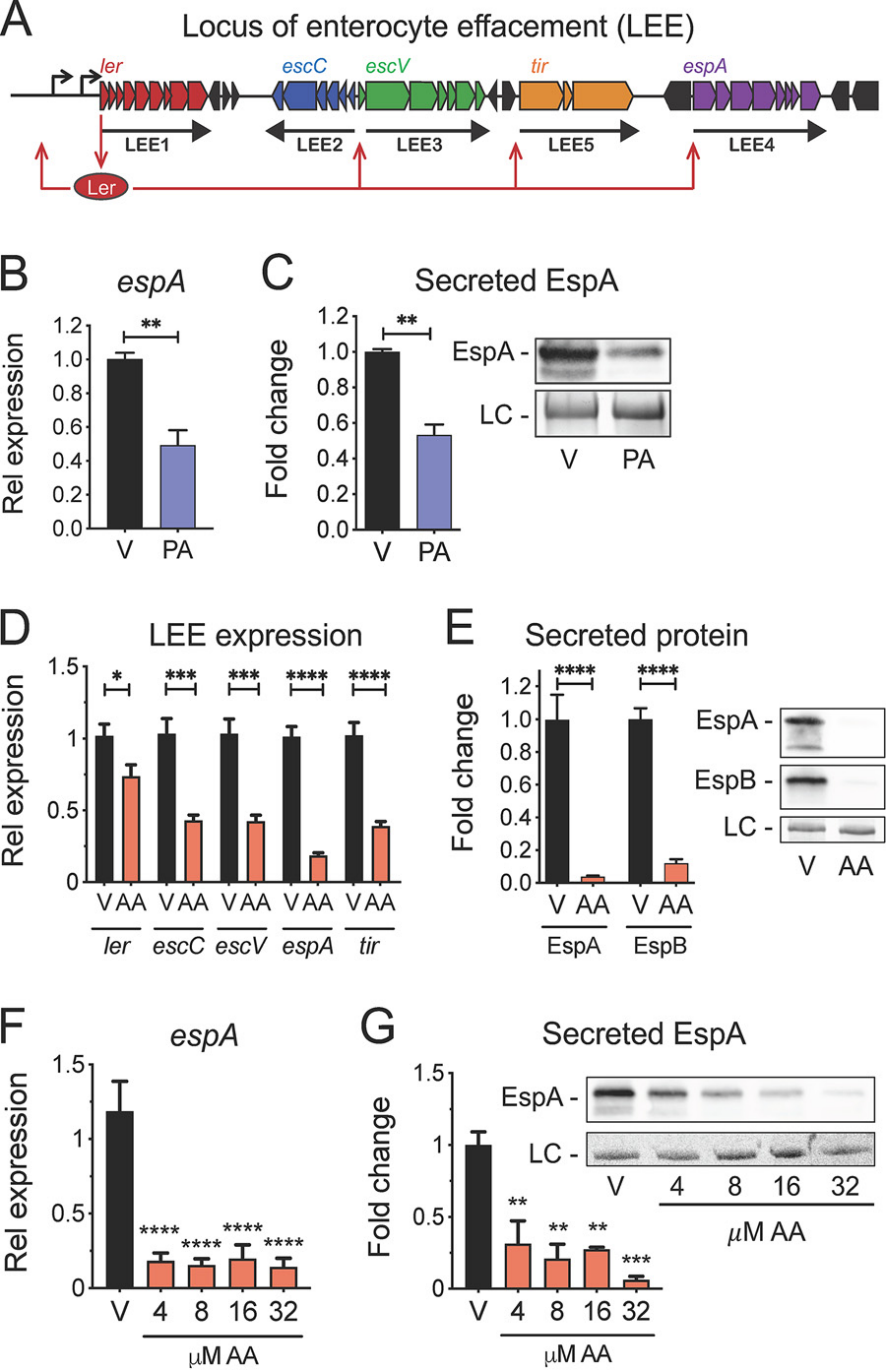
LCFAs inhibit the LEE pathogenicity island in EHEC. (A) Schematic of Ler regulation of the LEE pathogenicity island in EHEC. (B to E) EHEC was grown microaerobically under LEE-inducing conditions in the presence of 8 µM palmitic acid (PA), 8 µM arachidonic acid (AA), or the vehicle control (V). (B) Relative expression of the LEE-carried gene *espA* in EHEC as assessed by targeted qRT-PCR. (C) EHEC secretion of the LEE effector EspA at late log phase as assessed by Western blotting (right) and densitometry (left). (D) Relative expression of representative genes from each of the 5 LEE operons in EHEC as assessed by targeted qRT-PCR. (E) EHEC secretion of EspA and EspB at late log phase as assessed by Western blotting (right) and densitometry (left). (F) Relative expression of *espA* in EHEC in response to a range of arachidonic acid (AA) doses as assessed by targeted qRT-PCR. (G) EHEC secretion of EspA at late log phase in response to a range of arachidonic acid (AA) doses as assessed by Western blotting (right) and densitometry (left). LC, loading control. All data are represented as the mean ± SEM from at least 3 independent experiments. *P* values were determined by Student’s unpaired *t* test (B and D) Mann-Whitney test (C and E), one-way ANOVA (F), or Kruskal-Wallis test (G). *, *P* < 0.05; **, *P* < 0.01; ***, *P* < 0.001; ****, *P* < 0.0001.

A variety of signals present in the gut, including sugars, peptides, and lipids, are sensed by EHEC and are integrated into the complex intracellular signaling cascades that regulate the LEE (2, 7–11). Transcriptional activation of the LEE is regulated by the master transcription factor Ler, which is the first gene contained within *LEE1* (Fig. 1A) (12, 13). The nearly 1,000 bp that comprise the regulatory region upstream from the *LEE1* promoter are heavily trafficked by different transcription factors that each sense and respond to specific stimuli, thus enabling the direct coupling of environmental signals to the transcriptional regulation of *ler* and the entire LEE island (7, 9, 10, 14, 15).

Long-chain fatty acids (LCFAs) are nonesterified fatty acids that consist of 12 or more carbon atoms and can serve as nutrient sources or as building blocks for membrane biosynthesis in bacteria. In E. coli, extracellular LCFAs (C_12–18_) are transported across the outer membrane through FadL and are then thought to traverse the periplasm and flip across the inner membrane to its inner leaflet (16–19). Upon entering the cytosol, LCFAs are activated into long-chain acyl coenzyme A (acyl-CoA) thioesters by FadD (20). This enables both their stepwise breakdown by fatty acid degradation enzymes through the beta-oxidation cycle and the direct sensing of long-chain acyl-CoAs by the GntR family transcription factor FadR, which controls the expression of genes involved in fatty acid degradation (*fad*) and biosynthesis (*fab*) (21, 22). In its apo form, FadR binds its target DNA sequence, which results in the repression of *fad* genes and the activation of *fab* genes (23). In contrast, FadR binding of long-chain acyl-CoAs decreases its affinity for DNA, resulting in transcriptome-wide changes to the FadR regulon (23–26). Thus, FadR couples the sensing of intracellular LCFA pools with the regulation of lipid metabolism and utilization in E. coli. The contribution of LCFA metabolism to *in vivo* fitness has been described for numerous intestinal pathogens (27–32).

More recently, several studies have reported that exogenous LCFAs also act as signals that directly modulate virulence in enteric pathogens (33–38). In Salmonella enterica and Vibrio cholerae, free LCFAs directly block the activation of virulence genes through mechanisms that are independent of canonical LCFA processing by FadD and consequent sensing by FadR (35–39). In V. cholerae, unsaturated fatty acids (UFAs) such as oleic acid (C_18:1_) and linoleic acid (C_18:2_) directly bind the AraC-like provirulence regulator ToxT, which prevents ToxT binding to its DNA targets and consequent activation of downstream virulence genes (35, 37–39). Similarly, in S. enterica, free UFAs such as oleic acid (C_18:1_) and saturated fatty acids such as palmitic acid (C_16:0_) interact with HilD, the AraC-like master regulator of the SPI-1 pathogenicity island, which subsequently prevents HilD binding with its DNA targets and downstream activation of SPI-1 genes (36). In V. cholerae, a FadR-dependent mechanism has also been described for the regulation of ToxT-dependent virulence in the absence of exogenous LCFAs. This involves the positive regulation of ToxT levels by the fatty acid biosynthesis gene *fabA*, which is activated by FadR, through an undefined posttranslational mechanism (40). Similarly, we have also shown that FadR regulates the LEE pathogenicity island in EHEC in the absence of exogenously added LCFAs (15). Taken together, LCFAs (C_<20_) and the LCFA-CoA sensor FadR have been linked to the regulation of virulence in numerous enteric pathogens through distinct mechanisms.

Arachidonic acid (C_20:4_, *cis* 5,8,11,14) is an omega-6, polyunsaturated fatty acid (PUFA) that is uniquely present within mammalian, but not bacterial, membranes. In response to environmental stimuli, arachidonic acid is liberated from membrane-associated phospholipids and monoacylglycerols and acts as an important host signaling molecule that modulates a diverse range of host functions (41, 42). Arachidonic acid also exerts potent growth-inhibitory effects on numerous Gram-positive and Gram-negative pathogens through mechanisms that involve oxidative stress and perturbation of membrane integrity (43–46). In contrast, several *Enterobacteriaceae* species such as E. coli and Pseudomonas aeruginosa exhibit resistance to the bactericidal effects of arachidonic acid (43). However, the impact of arachidonic acid on bacterial function and virulence, including interactions with components of the canonical LCFA sensing machinery in E. coli, remains poorly understood. Because we previously identified FadR as a novel regulator of the LEE, we sought to investigate the effects of LCFAs on EHEC virulence with a specific focus on arachidonic acid. Here, we demonstrate that the LCFAs palmitic acid and arachidonic acid inhibit the activation of the LEE in EHEC in a FadR-dependent manner. Functional, biochemical, and genetic studies further revealed that similarly to shorter LCFAs (C_12–18_), arachidonic acid is activated by FadD and then, in its acyl-CoA form, serves as a signaling molecule that interacts with FadR. This interaction decreases FadR binding to its DNA targets within the *LEE1* promoter region, resulting in decreased transcriptional activation of the LEE and attenuated virulence. In addition to demonstrating the antivirulence effects of arachidonic acid, our findings also suggest that the canonical LCFA sensing system in EHEC recognizes LCFAs longer than the typical 18 carbons, which could expand the repertoire of LCFAs that are directly sensed by E. coli and other *Enterobacteriaceae*. More broadly, our findings also demonstrate that in addition to its established effects on host immune function and its bactericidal effects on certain pathogens, arachidonic acid also serves as a signaling molecule that directly modulates pathogen virulence and function.

## RESULTS

### Arachidonic acid inhibits the LEE pathogenicity island in EHEC.

The foodborne pathogen EHEC utilizes a T3SS encoded within the LEE pathogenicity island to successfully establish enteric infection. Most of the genes within the LEE are organized in 5 major operons (*LEE1* to *LEE5*). Transcriptional activation of the LEE is regulated by the master regulator Ler encoded within *LEE1* (Fig. 1A). We previously reported that the LCFA-CoA-responsive transcription factor, FadR, directly binds DNA targets located upstream from the *LEE1* promoter (15). We therefore sought to determine whether exogenous LCFAs impact EHEC virulence by transcriptionally modulating the LEE. To initially explore this, we first focused on palmitic acid, an LCFA that interacts with FadR when activated by the acyl-CoA synthetase (ACS) FadD (21, 47). The addition of micromolar concentrations of palmitate corresponded with decreased expression of the LEE gene *espA* (Fig. 1B), which encodes a secreted protein that forms a filament that wraps around the T3SS needle (48, 49). Similarly, we observed decreased secretion of EspA in the presence of palmitate when EHEC was cultivated under *in vitro* conditions known to activate the LEE (Fig. 1C) (50, 51). Together, these initial investigations suggest that the LCFA palmitate inhibits the LEE in EHEC.

To further explore the effects of LCFAs on EHEC virulence, we next focused on arachidonic acid, a PUFA that is ubiquitous within mammalian membrane phospholipids and is present in its liberated form in the mammalian gut during enteric infection (11). In the presence of arachidonic acid, the transcription of representative genes from each of the five LEE operons was significantly decreased (Fig. 1D). This corresponded with the reduced functionality of the LEE-encoded T3SS system as assessed by secretion of the translocon components EspA and EspB (Fig. 1E; see also Fig. S1A in the supplemental material). The transcriptional and functional repression of the LEE was observed over a range of physiological concentrations of arachidonic acid (Fig. 1F and G) (41). Importantly, arachidonic acid did not appear to exert an overall antivirulence effect on EHEC because the transcription of the phage-encoded virulence factor Shiga toxin, which causes hemolytic-uremic syndrome (52), was not altered (Fig. S1B). Taken together, in addition to palmitate, the LCFA arachidonic acid also exerts inhibitory effects on the LEE in EHEC.

10.1128/mBio.03247-20.1 FIG S1 The effects of arachidonic acid on the LEE and Shiga toxin in EHEC. EHEC was grown microaerobically under LEE-inducing conditions in the presence of 8 µM arachidonic acid (AA) or the vehicle control (V). (A) Secretion profile of proteins in EHEC at late log phase. (B) Relative expression of the phage-carried gene *stx2a* in EHEC as assessed by targeted qRT-PCR. LC, loading control. All data are represented as the mean ± SEM from at least 3 independent experiments. *P* values were determined by Student’s unpaired *t* test. Download FIG S1, PDF file, 0.1 MB. Copyright © 2021 Ellermann et al. 2021 Ellermann et al. This content is distributed under the terms of the Creative Commons Attribution 4.0 International license.

### Inhibition of the LEE by arachidonic acid is not dependent on EHEC growth.

**FIG 2:**
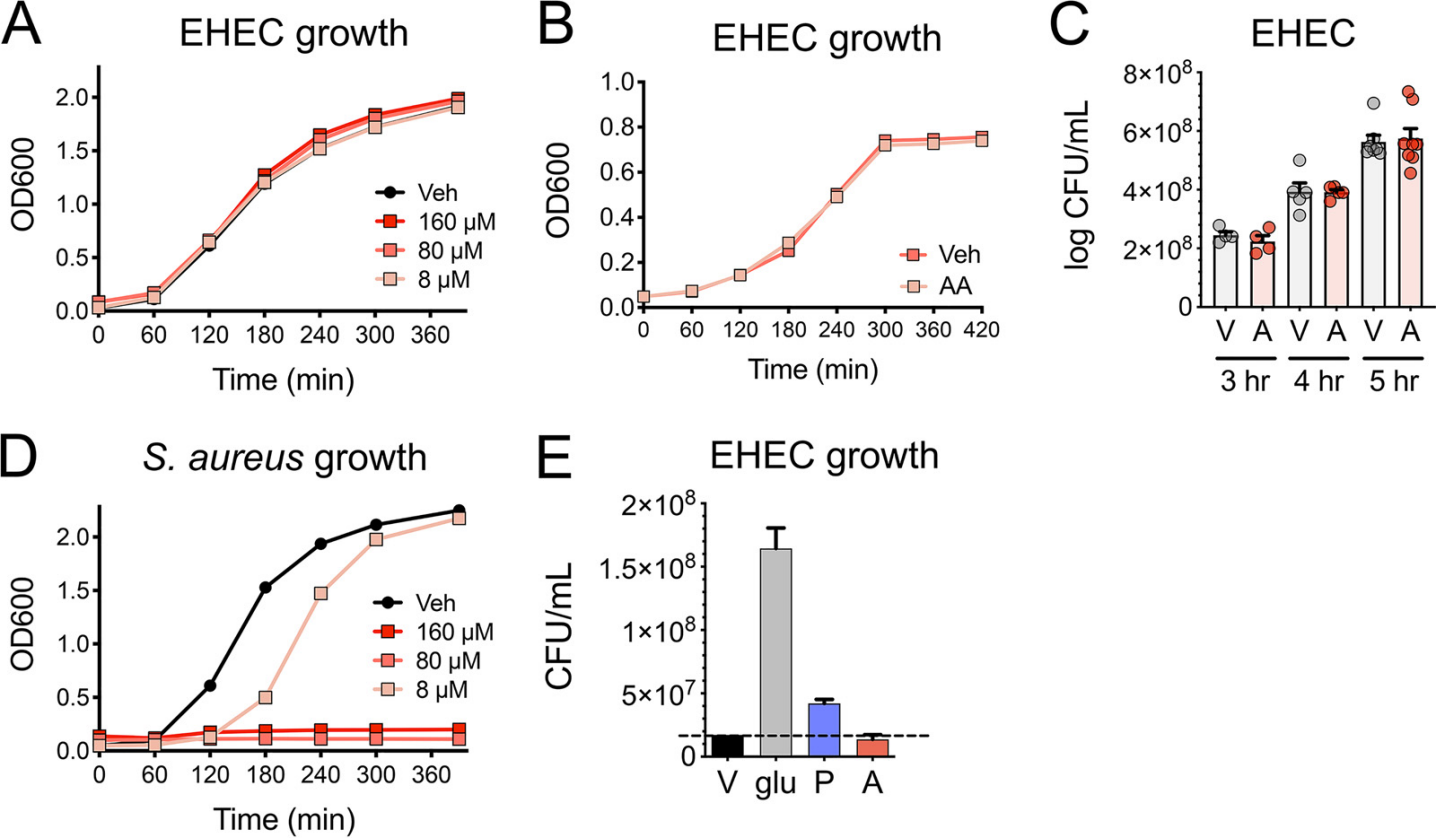
Arachidonic acid does not impact EHEC growth. (A) Aerobic growth kinetics of EHEC in LB medium with the indicated concentrations of arachidonic acid or vehicle control (Veh). (B and C) EHEC was grown microaerobically under LEE-inducing conditions in the presence of 8 µM arachidonic acid (A) or the vehicle control (V). (B) Microaerobic growth kinetics of EHEC. (C) Quantitative culture of EHEC at the indicated time points. (D) Aerobic growth kinetics of S. aureus in brain heart infusion (BHI) medium with the indicated concentrations of arachidonic acid or vehicle control. (E) Quantitative culture of EHEC grown microaerobically in minimal medium in the absence of a carbon source (V) or in the presence of glucose (glu), 8 80 µM palmitic acid (P), or 1.2 mM arachidonic acid (A) as sole carbon source. All data are represented as the mean ± SEM from at least 3 independent experiments. The dashed horizontal line represents the CFU/ml of EHEC recovered without a carbon source.

Arachidonic acid has been reported to be a potent antibacterial compound against numerous bacterial pathogens at micromolar concentrations (43–46). Therefore, we next determined whether the antivirulence effects of arachidonic acid in EHEC occur through a bacteriostatic or bactericidal mechanism. To accomplish this, we cultivated EHEC under microaerobic and aerobic conditions in the presence of physiological concentrations of arachidonic acid and did not observe any growth defects (Fig. 2A to C). In contrast, as previously reported, similar concentrations of arachidonic acid exerted growth-inhibitory effects on the Gram-positive organism Staphylococcus aureus (Fig. 2D) (43). LCFAs such as palmitic acid also serve as carbon sources for E. coli through the beta-oxidation metabolic pathway (21). We therefore investigated whether EHEC can utilize arachidonic acid as a nutrient source, which in turn may contribute to the inhibitory effects of arachidonic acid on the LEE. To accomplish this, we tested whether EHEC can grow in a defined minimal medium with arachidonic acid as the sole carbon source. While EHEC can utilize glucose and palmitic acid as sole carbon sources, EHEC failed to replicate in the presence of arachidonic acid as a sole carbon source (Fig. 2E). Taken together, these results suggest that the inhibitory effects of arachidonic acid on the LEE are unlikely to be due to any detrimental or promotional effects on EHEC growth.

### Repression of the LEE in the presence of arachidonic acid is dependent on *fadR*.

**FIG 3:**
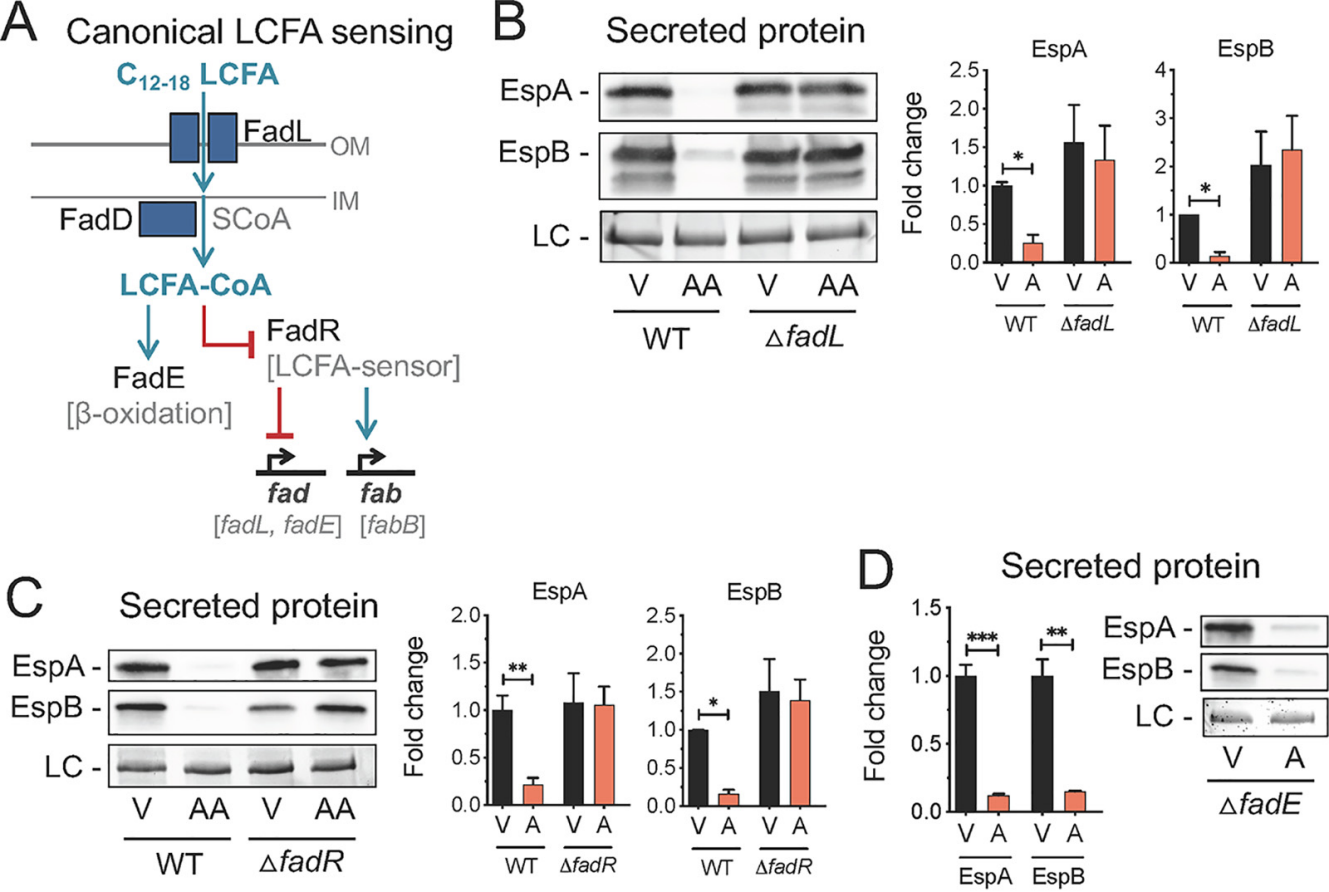
Inhibition of the LEE by arachidonic acid is dependent on FadR. (A) Schematic of canonical long-chain fatty acid (LCFA) sensing in Escherichia coli. (B to D) EHEC was grown microaerobically under LEE-inducing conditions in the presence of 8 µM arachidonic acid (AA) or the vehicle control (V). (B) Secretion of the LEE effectors EspA and EspB at late log phase by EHEC WT or Δ*fadL* strain as assessed by Western blotting (left) and densitometry (right). (C) Secretion of EspA and EspB by EHEC WT or Δ*fadR* strain as assessed by Western blotting (left) and densitometry (right). (D) Secretion of EspA and EspB by EHEC WT or Δ*fadE* strain as assessed by Western blotting (right) and densitometry (left). LC, loading control. All data are represented as the mean ± SEM from at least 3 biological replicates. *P* values were determined by Kruskal-Wallis test. *, *P* < 0.05; **, *P* < 0.01; ***, *P* < 0.001.

In *Enterobacteriaceae*, fluctuations in intracellular LCFAs are sensed by the FadR transcriptional regulator (24). Exogenous LCFAs can also be sensed by FadR following transport across the outer membrane through FadL (Fig. 3A) (21, 47). We next investigated whether components of this LCFA sensing pathway are required for arachidonic acid to exert its inhibitory effects on the LEE. We first constructed a *fadL* isogenic mutant in EHEC and investigated the effects of arachidonic acid on LEE-dependent T3SS activity in this mutant. In contrast to the parental strain, arachidonic acid did not alter secretion of EspA and EspB or protein expression of EspA and the LEE effector Tir in the *fadL* mutant (Fig. 3B and Fig. S2A). Next, we assessed whether LEE activity is altered in a *fadR* isogenic mutant in EHEC in response to arachidonic acid. As with the *fadL* mutant, arachidonic acid failed to repress the LEE in the *fadR* mutant (Fig. 3C and Fig. S2B). Similarly, both *fadL* and *fadR* are required to mediate the inhibitory effects of palmitic acid on the LEE in EHEC (Fig. S2C). Finally, to further rule out the possibility that the beta-oxidation pathway contributes to the antivirulence effects of arachidonic acid, we also tested the effects of arachidonic acid on LEE-dependent T3SS activity in the isogenic *fadE* mutant, which is deficient in the first enzyme that initiates beta-oxidation (Fig. 3A) (53). As with the parental strain, the *fadE* mutant also exhibited decreased EspA and EspB secretion in response to palmitic acid and arachidonic acid (Fig. 3D). Importantly, no growth effects were observed in the *fadL*, *fadR*, or *fadE* isogenic mutants under control conditions or in the presence of arachidonic acid (Fig. S2D and E). Taken together, our genetic studies demonstrate that both *fadL* and *fadR* are required for arachidonic acid and palmitic acid to exert their antivirulence effects on the LEE in EHEC through a mechanism that is not dependent on fatty acid metabolism.

10.1128/mBio.03247-20.2 FIG S2 Inhibition of the LEE by LCFAs is dependent on FadR. EHEC was grown microaerobically under LEE-inducing conditions in the presence of 8 µM arachidonic acid (AA), 8 µM palmitic acid (PA), or the vehicle control (V). (A) EspA and Tir in whole-cell lysates at late log phase from EHEC WT or Δ*fadL* strain as assessed by Western blotting. (B) EspA and Tir in whole-cell lysates at late log phase from EHEC WT or Δ*fadR* strain as assessed by Western blotting. (C) Secretion of the LEE effector EspA at late log phase by EHEC WT or its isogenic mutants as assessed by Western blotting. (D and E) Microaerobic growth kinetics of EHEC or its isogenic mutants in the presence of the vehicle control (D) or arachidonic acid (E). Download FIG S2, PDF file, 0.4 MB. Copyright © 2021 Ellermann et al. 2021 Ellermann et al. This content is distributed under the terms of the Creative Commons Attribution 4.0 International license.

### Arachidonic acid is processed by FadD to enable direct interaction with FadR.

**FIG 4:**
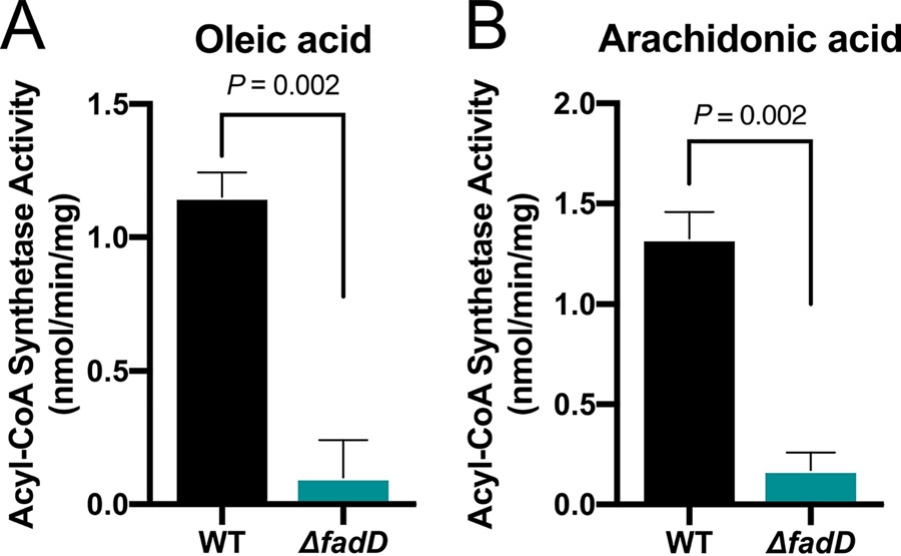
FadD catalyzes the production of arachidonoyl-CoA in EHEC. *In vitro* acyl-CoA synthesis activity assay with EHEC WT or Δ*fadD* bacterial lysates using 20 µM oleic acid (A) or 20 µM arachidonic acid (B) as the substrates. All data are represented as the mean ± SEM from 6 biological replicates. *P* values were determined by Mann-Whitney test.

**FIG 5:**
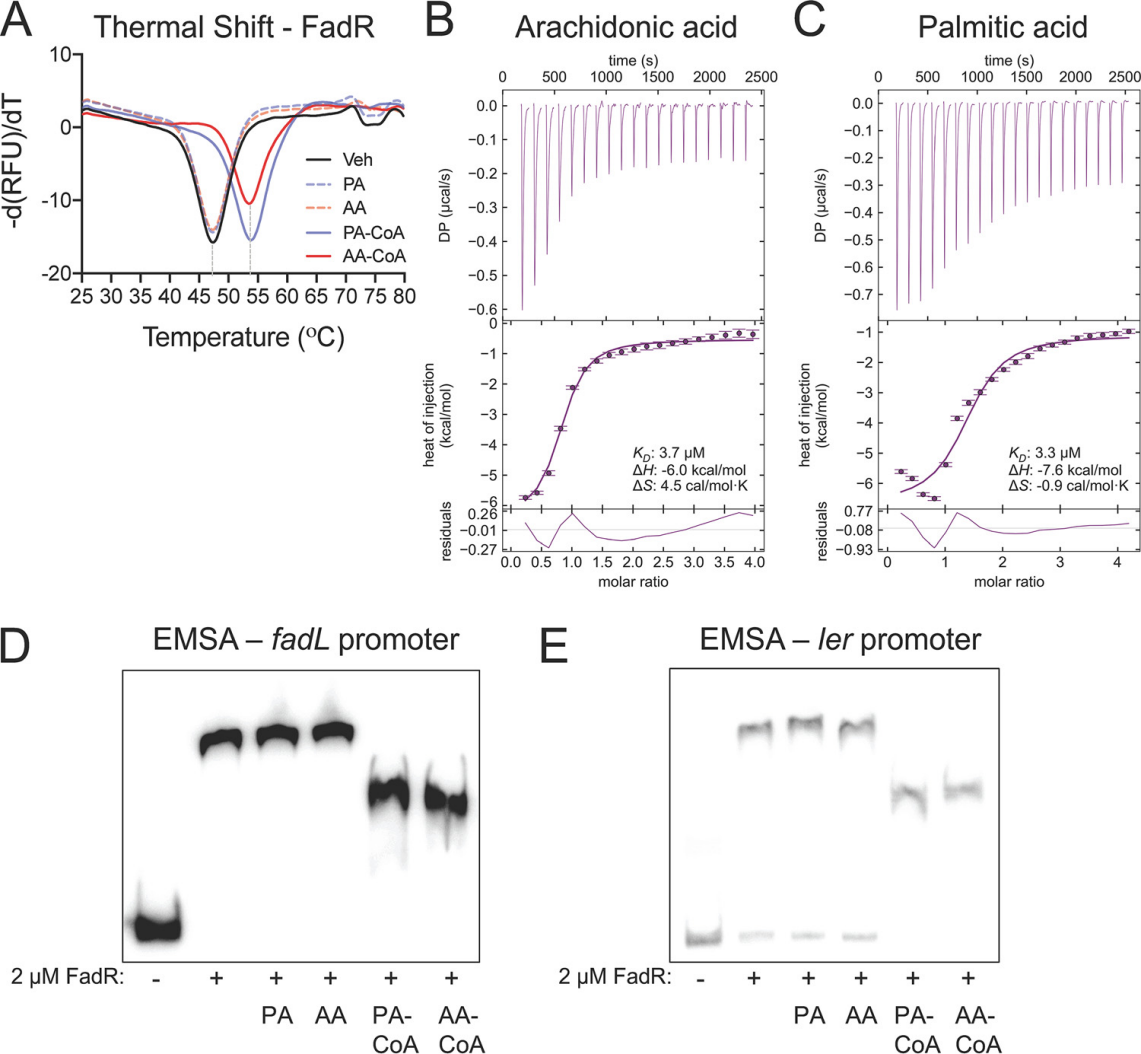
Arachidonoyl-CoA directly interacts with FadR to inhibit binding at its DNA targets. (A) Thermal unfolding of recombinant FadR is monitored using SYPRO Orange. Data were collected in the presence or absence of the indicated long-chain fatty acid (LCFA) or acyl-CoA at 20 µM, leading to a rightward shift in the unfolding transition. All data depict representative curves from 3 independent experiments with 3 technical replicates. (B and C) Isothermal titration calorimetry (ITC) isotherms of the EHEC FadR protein with approximately 0.5 mM arachidonoyl-CoA (AA-CoA) (B) or palmitoyl-CoA (PA-CoA) (C) at 20°C. The raw thermogram of each experiment is shown. The lower panel in panels B and C indicates the titration curve fitted to the one-site model. Residuals between the data and the fit lines are shown in the lowest plot. All data were integrated using NITPIC and analyzed in SEDPHAT. (D and E) Electrophoretic mobility shift assays (EMSAs) for EHEC FadR in the presence of the LCFAs palmitic acid (PA) and arachidonic acid (AA) or their respective acyl-CoAs at 20 µM using the FadR binding sites within the *fadL* (D) and *ler* (E) promoters. All images are representative of 2 independent experiments.

In E. coli, FadR senses intracellular levels of LCFAs by binding to their acyl-CoA analogs that are generated by FadD (Fig. 3A) (25, 26, 54). To our knowledge, the activation of LCFA substrates longer than 18 carbons by E. coli FadD has not been described. Therefore, we sought to determine whether FadD in EHEC can utilize arachidonic acid as a substrate for acyl-CoA synthetase activity. As reported with oleic acid (25), acyl-CoA synthetase activity was observed when free arachidonic acid was added as a substrate to EHEC lysates (Fig. 4). In contrast, minimal acyl-CoA synthetase activity was detected with the addition of oleic acid or arachidonic acid to *fadD* mutant lysates. These data suggest EHEC can activate arachidonic acid through FadD acyl-CoA synthetase activity, which could then enable consequent interactions with FadR. Next, we determined whether EHEC FadR can directly interact with arachidonic acid-CoA (AA-CoA) *in vitro*. When a protein binds its ligand, the thermal stability of the protein increases, which can be detected by a shift in the melting temperature when performing fluorescent thermal shift assays. Therefore, we utilized this technique to initially establish whether purified FadR from EHEC can interact with AA-CoA, using palmitic acid-CoA (PA-CoA) as a positive control. We observed that the thermal stability of FadR was increased in the presence of PA-CoA or AA-CoA (Fig. 5A). In contrast, the addition of free palmitic acid or arachidonic acid did not alter the thermal stability of FadR, suggesting that FadR interacts with the acyl-CoA analogs of palmitic acid and arachidonic acid. To further confirm that purified FadR from EHEC can directly interact with AA-CoA, we performed isothermal calorimetry with FadR. We observed that the equilibrium disassociation constants for AA-CoA and PA-CoA were comparable (AA-CoA: *K_D_* = 3.7 µM; PA-CoA: *K_D_* = 3.3 µM) (Fig. 5B and C), suggesting that FadR binds the two acyl-CoAs with similar binding affinities. Together, these data demonstrate that FadR directly interacts with AA-CoA, which in turn may modulate FadR binding to its DNA targets, including binding sequences located within the *LEE1* promoter regulatory region.

### AA-CoA limits the interaction of FadR with its canonical DNA targets.

In its apo form, FadR binds its DNA targets to repress *fad* genes and to activate *fab* genes (23). FadR binding to acyl CoAs diminishes its affinity for DNA, simultaneously alleviating its transcriptional repression of *fad* genes and its activating effects on *fab* genes (Fig. 3A). We therefore investigated whether AA-CoA also modulates FadR binding with its canonical DNA targets. To accomplish this, we first conducted thermal stability assays in the presence of purified oligonucleotides containing the FadR binding motif within the *fadL* promoter. We observed that FadR interacts with the *fadL* probe as expected, which is indicated by a positive shift in the melting temperature (Fig. S3A). In comparison, a further positive shift in the melting temperature is apparent when either PA-CoA or AA-CoA is added in the absence of the *fadL* probe (Fig. S3B). This suggests that when FadR interacts with its acyl-CoA ligands, its thermal stability increases compared to its target DNA sequences. When PA-CoA or AA-CoA was added in the presence of the *fadL* promoter, further thermal stabilization of FadR was not apparent beyond that observed with the addition of the acyl-CoA ligands alone (Fig. S3B). Together, these observations suggest that similar to PA-CoA, AA-CoA can interact with FadR, which reduces its affinity for its DNA target. To further demonstrate that AA-CoA modulates FadR binding to its canonical DNA targets, we conducted electrophoretic mobility shift assays (EMSAs) on previously published FadR binding sites for the *fadL* promoter in EHEC (15). In the absence of the acyl-CoA ligands or in the presence of free LCFAs, a shift in the *fadL* probe was observed, which is consistent with the FadR binding to DNA in its apo form. In contrast, upon the addition of the FadR ligand PA-CoA or AA-CoA, the shift of the *fadL* probe was decreased, which indicates diminished FadR interactions with its binding motif (Fig. 5D). Importantly FadR in its apo form did not cause a shift when using a *kan* promoter probe as a negative control (Fig. S3C). Taken together, these data demonstrate that as has been previously established with PA-CoA, AA-CoA also modulates FadR interactions with its target canonical DNA sequences.

10.1128/mBio.03247-20.3 FIG S3 Inhibition of the LEE by LCFAs is dependent on FadR. Thermal unfolding of FadR is monitored using SYPRO Orange. (A) Data were collected in the absence (control) or presence of DNA (*fadL* promoter sequence with the FadR binding site). (B) Data were collected with FadR alone, in the presence of DNA containing the FadR binding site within the *fadL* promoter (binding site), in the presence of 20 µM acyl-CoAs (palCoA or AACoA), or in the presence of DNA with acyl-CoAs. (C) Electrophoretic mobility shift assays (EMSAs) for EHEC FadR using the *kan* promoter as a probe to serve as a negative control. Download FIG S3, PDF file, 0.2 MB. Copyright © 2021 Ellermann et al. 2021 Ellermann et al. This content is distributed under the terms of the Creative Commons Attribution 4.0 International license.

### AA-CoA interactions with FadR modulate its regulation of the LEE.

**FIG 6:**
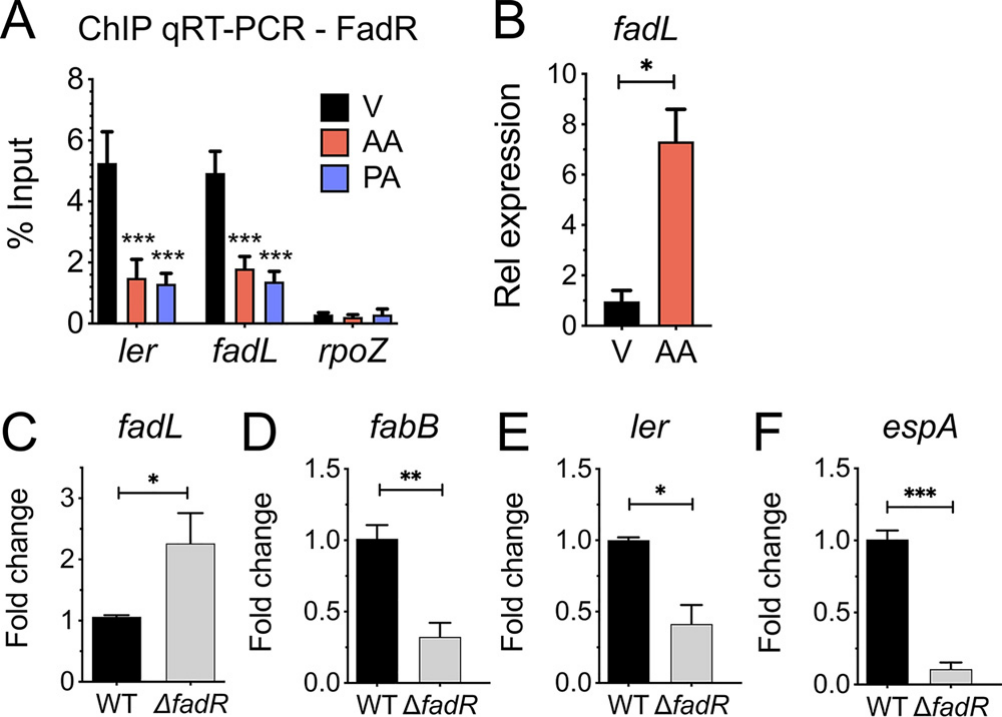
FadR acts as a transcriptional activator of the LEE. (A and B) EHEC was grown microaerobically under LEE-inducing conditions in the presence of 8 µM arachidonic acid (AA), 8 µM palmitic acid (PA), or the vehicle control (V). (A) ChIP-qPCR of N-terminally tagged FadR. Probes are designed to amplify the promoter regions of *fadL* (positive control), *rpoZ* (negative control), or *ler* (*LEE1*). Data are displayed as percentages of the protein input. (B) Relative expression of *fadL* as assessed by targeted qRT-PCR. (C to F) EHEC WT or Δ*fadR* strain was grown aerobically in LB medium. Relative expression of *fadL* (C), *fabB* (D), *ler* (E), or *espA* (F) in EHEC WT or Δ*fadR* strain at late log growth phase as assessed by qRT-PCR. All data are represented as the mean ± SEM from 3 biological replicates. *P* values were determined by two-way ANOVA (A) or Student’s unpaired *t* test (B to F). *, *P* < 0.05; **, *P* < 0.01; ***, *P* < 0.001.

We have previously shown that FadR interacts with target DNA sequences located within the regulatory region upstream from the *LEE1* promoter (15). Therefore, we next sought to demonstrate whether AA-CoA impacts FadR-DNA interactions at the *LEE1* promoter *in vitro*. To accomplish this, we first conducted EMSAs on the previously published FadR distal binding sites upstream from the *LEE1* promoter in EHEC (15). As we have previously reported, addition of FadR protein alone resulted in a shift of the *LEE1* promoter probe. In contrast, as observed with the *fadL* promoter probe, addition of AA-CoA or PA-CoA decreased the ability of FadR to shift the *LEE1* promoter probe (Fig. 5E). We next addressed whether AA-CoA can modulate FadR activity *in vivo* by conducting chromatin immunoprecipitation coupled with quantitative PCRs (ChIP-qPCR) at the *fadL* and *LEE1* promoters. In agreement with our EMSAs, we observed a significant decrease in FadR recovered from the *ler* and *fadL* promoters upon the addition of AA or PA compared to the vehicle control (Fig. 6A). This corresponded with increased expression of *fadL* (Fig. 6B) and decreased expression of *ler* in the presence of arachidonic acid (Fig. 1D). Taken together, these findings support our hypothesis that AA-CoA modulates FadR binding at the *LEE1* promoter.

### FadR acts as a transcriptional regulator of the LEE.

Because we have established that FadR can directly interact with AA-CoA, we hypothesized that arachidonic acid represses the LEE by modulating FadR binding at the *LEE1* promoter. Given that FadR binds DNA in the absence of acyl-CoA ligands, we predicted that under microaerophilic and aerobic conditions FadR acts as a transcriptional activator of the LEE. This hypothesis is further supported by the increased expression of *fadL*—a gene that has been established to be repressed by FadR—in the presence of arachidonic acid (Fig. 6B). To further investigate this hypothesis, we compared the expression levels of representative genes from the canonical FadR regulon and the LEE in EHEC wild type (WT) and the *fadR* mutant at late log phase under aerobic conditions when *fadR* is maximally expressed (Fig. S4A). As has been previously reported for E. coli, *fadL* expression is increased and *fabB* expression is decreased in the *fadR* mutant compared to the parental strain (Fig. 6C and D) (23). We also observed that the expression of the LEE genes *ler* and *espA* is decreased in the *fadR* mutant, which is similar to the transcriptional expression patterns we observed with the FadR-activated gene *fabB*. We performed similar expression studies with EHEC cells harvested during exponential growth (mid-log phase). In contrast to late log phase, we observed that *fadL* expression is decreased and *fabB*, *ler*, and *espA* expression is increased in the *fadR* mutant (Fig. S4B to E), which may be indicative of altered intracellular LCFA content in mid- versus late log phase in the absence of exogenously added LCFAs. Nonetheless, as observed in late log phase, the expression patterns of *ler* and *espA* corresponded to that of *fabB* during exponential growth, further supporting our observation that FadR exerts similar regulatory effects at the *LEE1* and *fabB* promoters. Taken together, our findings are consistent with our hypothesis that FadR can act as a transcriptional activator at the *LEE1* promoter.

10.1128/mBio.03247-20.4 FIG S4 Expression patterns of the FadR regulon in EHEC WT versus Δ*fadR* mutant at mid-log growth. EHEC WT or Δ*fadR* strain was grown aerobically in LB medium. Relative expression of *fadR* in EHEC WT (A) and *fadL* (B), *fabB* (C), *ler* (D), or *espA* (E) in EHEC WT or Δ*fadR* at mid-log growth phase. All data are represented as the mean ± SEM from 3 biological replicates. *P* values were determined by Student’s unpaired *t* test. *, *P* < 0.05; **, *P* < 0.01; ***, *P* < 0.001. Download FIG S4, PDF file, 0.1 MB. Copyright © 2021 Ellermann et al. 2021 Ellermann et al. This content is distributed under the terms of the Creative Commons Attribution 4.0 International license.

### Arachidonic acid inhibits EHEC infection and lesion formation on epithelial cells in a FadR-dependent manner.

**FIG 7:**
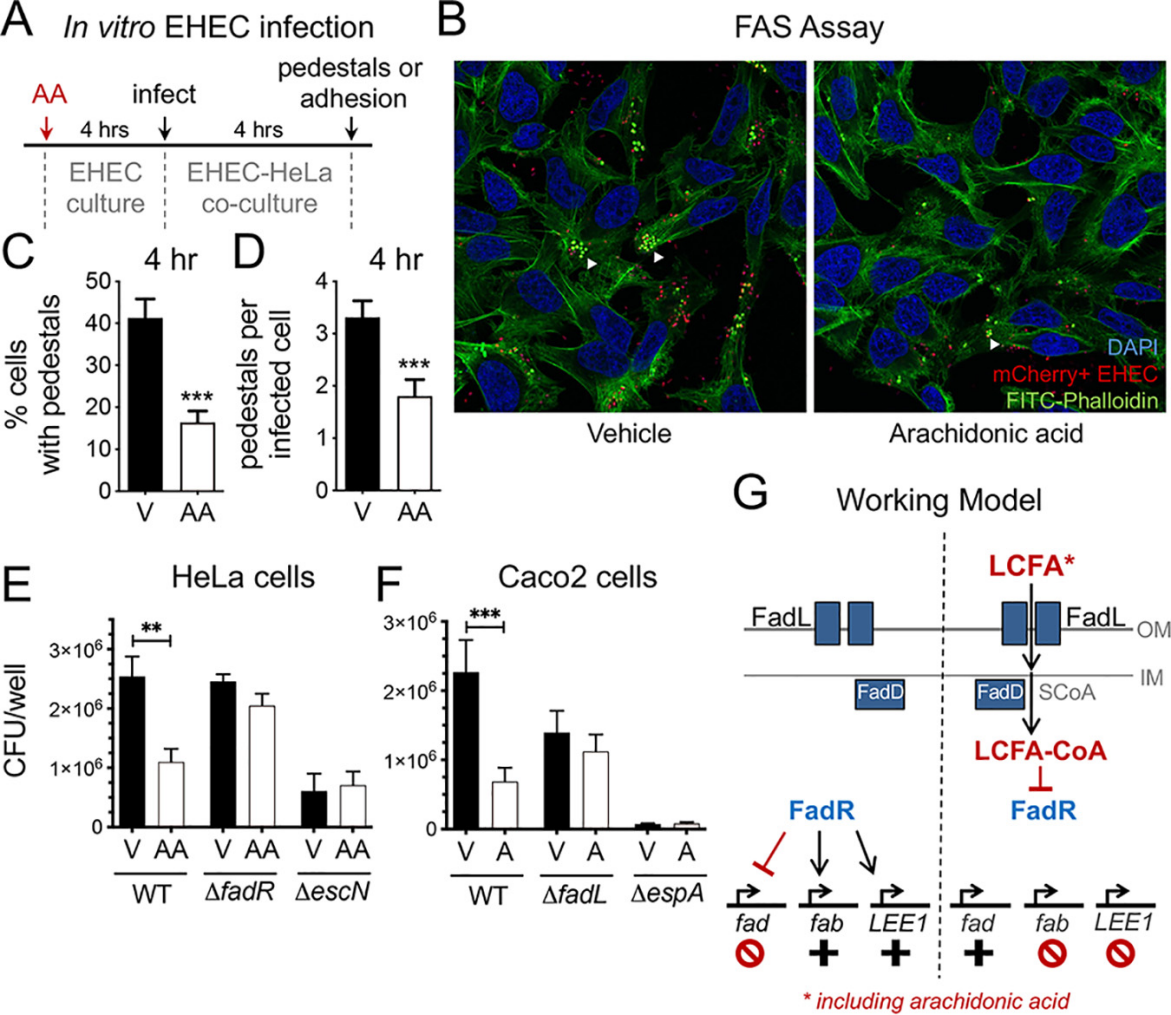
Arachidonic acid inhibits epithelial EHEC infection in a FadR-dependent manner. (A) Schematic of EHEC *in vitro* infection model. (B to E) EHEC was grown microaerobically under LEE-inducing conditions (DMEM-low glucose) with 8 µM arachidonic acid (AA) or the vehicle control (V). At late log phase, arachidonic acid- or vehicle-treated EHEC was transferred to a coculture system with HeLa cells to initiate EHEC infection. (B) Representative confocal microscopy images of LEE-dependent pedestal formation (white arrowheads) on epithelial cells by mCherry-expressing EHEC. DNA (blue) is stained with DAPI, and actin (green) is stained with FITC-phalloidin. Images at 40×. (C) Percentage of epithelial cells infected with EHEC pedestals at 4 h postinfection. (D) Quantity of EHEC pedestals per infected epithelial cell at 4 h postinfection. At least 275 cells in 17 fields at 40× were enumerated for each group. (E) Quantitative culture of EHEC WT and its isogenic mutants recovered from an adhesion assay with HeLa cells at 4 h postinfection. (F) EHEC was grown microaerobically under LEE-inducing conditions (DMEM-low glucose) with arachidonic acid (A) or the vehicle control (V). At late log phase, arachidonic acid- or vehicle-treated EHEC was transferred to a coculture system with Caco-2 cells to initiate EHEC infection. Quantitative culture of EHEC and its isogenic mutants recovered from an adhesion assay with Caco-2 monolayers at 5 h postinfection. (G) Schematic of model depicting long-chain fatty acid (LCFA) regulation of EHEC virulence and the canonical FadR regulon. All data are represented as the mean ± SEM from 3 independent experiments. *P* values were determined by Mann-Whitney test (C and D) or one-way ANOVA (E and F). **, *P* < 0.01; ***, *P* < 0.001.

A hallmark of EHEC infection is the formation of attaching and effacing lesions (i.e., pedestals) that requires the activation of the LEE pathogenicity island and the functionality of the LEE-encoded T3SS. To demonstrate that arachidonic acid can inhibit EHEC infection and pedestal formation, we first pretreated EHEC with arachidonic acid or the vehicle control and then infected epithelial cells to observe pedestal formation by confocal microscopy (Fig. 7A). We observed that pedestal formation was reduced by approximately 50% with AA-treated EHEC as assessed by the infection rate (Fig. 7C) and the number of pedestals per infected cell (Fig. 7B and D). We confirmed these findings using an epithelial adhesion assay, where we compared the quantity of attached EHEC to epithelial cells following pretreatment with the vehicle or arachidonic acid. As observed by microscopy, there was approximately 50% less EHEC attached to epithelial cells following treatment with arachidonic acid (Fig. 7E). The quantity of attached AA-treated EHEC was comparable to the attachment rate of the LEE-inactivated *escN* mutant. Importantly, pretreatment with arachidonic acid did not diminish epithelial attachment by the *fadR* mutant, further supporting our model that arachidonic acid sensing by FadR results in the repression of the LEE (Fig. 7E). We also performed adhesion assays in the Caco-2 colon cancer cell line and observed similar results (Fig. 7F). Taken together, our findings support a model where arachidonic acid is recognized by the canonical LCFA processing and sensing machinery in EHEC (Fig. 7G). This ultimately diminishes DNA binding by FadR, thus attenuating its ability to transcriptionally activate the *LEE1* operon including the master LEE activator *ler*, which results in the repression of the LEE pathogenicity island. More broadly, these findings demonstrate that in addition to its known growth-inhibitory effects reported for other pathogens, arachidonic acid also acts a signal that modulates the expression of canonical genes involved in LCFA metabolism and catabolism and that exerts antivirulence effects in EHEC.

## DISCUSSION

Polyunsaturated fatty acids (PUFAs) play important and complex roles in host immunity by directly acting as signaling molecules recognized by host cells and by serving as precursors for other bioactive lipids with proinflammatory or wound healing functions. Manipulation of lipid content in host tissues, including omega-6 PUFAs such as arachidonic acid (C_20:4_) and omega-3 PUFAs such as alpha-linoleic acid (C_18:3_), eicosapentaenoic acid (C_20:5_), and docosahexaenoic acid (C_22:6_), through dietary, genetic, or pharmacological interventions is associated with altered severity of disease in numerous experimental models of colitis (55, 56). In the context of enteric bacterial infection, the administration of different dietary oil supplements can alter disease outcomes in mice challenged with Citrobacter rodentium, a murine pathogen that serves as an established model for human EHEC and enteropathogenic E. coli (EPEC) infection (57–61). Given the high fat content and increased omega-6-to-omega-3 ratios characteristic of the Western diet, there is much interest in defining the molecular mechanisms by which specific PUFAs may modulate the outcomes of intestinal diseases such as enteric infection. However, much of the work to date has predominantly focused on the effects of PUFAs on the host response. As a result, far less is known about whether invading pathogens can directly sense host-derived and dietary lipids in the gut and how this local biochemical information may be incorporated into the regulation of their virulence programs. Our work introduces a defined molecular interaction by which LCFAs including arachidonic acid, a host-derived and dietary PUFA, can impact the outcome of infectious disease with the human enteric pathogen EHEC. Our work shows that LCFAs including palmitic acid and arachidonic acid act as signaling molecules that directly suppress the LEE pathogenicity island in EHEC, which contains genes that are essential for causing disease in the host. Moreover, our findings expand the repertoire of ligands sensed by the canonical LFCA sensing machinery in EHEC to include arachidonic acid. Our data support a model where arachidonic acid is activated by the canonical LCFA processing machinery in EHEC, enabling recognition by the LCFA-CoA-responsive transcription factor FadR. This interaction precludes FadR binding to its target DNA sequences within the *LEE1* promoter, which results in attenuated LEE transcription, T3SS function, and EHEC virulence. Thus, in addition to its established effects on host immunity and its bactericidal properties against other bacteria, our work demonstrates that arachidonic acid also directly inhibits pathogen virulence.

Arachidonic acid has mostly been characterized as a PUFA that exerts inhibitory effects on bacterial growth (43–46). Shorter-chain LCFAs such as oleic acid (C_18:1_), linoleic acid (C_18:2_), and palmitic acid (C_16:0_) in their free form have all been shown to inhibit virulence in enteric pathogens through distinct FadD- and FadR-independent mechanisms (34–39). Interestingly, one group reported that unsaturated fatty acids (UFAs) such as oleic acid and arachidonic acid can inhibit cholera toxin production in V. cholerae in a FadR-independent manner (34). Further mechanistic studies with oleic acid revealed that the provirulence transcription factor ToxT binds free oleic acid, thus preventing ToxT binding to DNA (35, 37–39). Presumably, a similar mechanism occurs with arachidonic acid-mediated repression of cholera toxin production.

Our work here establishes that LCFAs including palmitic acid and arachidonic acid modulate the virulence potential of a second enteric pathogen, EHEC, through a distinct, FadR-dependent mechanism defined by FadR binding of the acyl-CoA analogs of the LCFAs, resulting in the direct modulation of virulence gene expression and pathogen function. Our data from the secretion assays seem to indicate that arachidonic acid may have a more potent effect in inhibiting the LEE than palmitic acid. These observations could be explained by numerous reasons, including the poorer solubility of palmitic acid than of arachidonic acid and the ability of EHEC to utilize palmitic acid, and not arachidonic acid, as a substrate for fatty acid metabolism. Based on our biochemical studies investigating FadD and FadR interactions with the acyl-CoA analogs of palmitic acid and arachidonic acid, we have not observed any evidence to suggest that components of the FadR system exhibit higher affinity for arachidonic acid than for palmitic acid. Thus, it is likely that the apparent differences in the antivirulence effects of arachidonic acid versus palmitic acid may be the result of differences in their bioavailability. Therefore, future studies quantifying the relative abundances of free LCFAs in the intestinal lumen and at the epithelial interface during the course of EHEC infection are clearly warranted to investigate how local concentrations of these LCFAs correlate with LEE activity.

Our work previously identified FadR as a transcriptional regulator that binds DNA targets upstream from the *LEE1* promoter in EHEC and C. rodentium (15). Under *in vitro* anaerobic growth, genetic ablation of *fadR* enhanced the expression of LEE-containing genes in the absence of exogenous LCFAs, which initially suggested that FadR may act as a repressor of the LEE (15). However, our follow-up studies presented here demonstrate that the effects of *fadR* deletion on LEE expression are dependent on growth phase (Fig. 6). These contrasting effects of *fadR* deficiency on the LEE could be due to a variety of factors including fluctuations in intracellular concentrations of LCFAs as a result of changing metabolic states that occur throughout growth, lipid flux that occurs with changes in membrane phospholipid composition, and the direct effects of FadR on LCFA biosynthesis, transport, and metabolism. Moreover, oxygenation directly affects the expression of *fad* genes via the oxygen-sensitive ArcAB two-component system (62), which could further explain the differing effects of *fadR* deletion on the LEE under anaerobic versus microaerobic conditions. Thus, to clarify the function of FadR as a transcriptional activator or repressor of the LEE, we performed a series of biochemical and functional studies to investigate the effects of long-chain acyl-CoA ligands on FadR DNA binding and LEE expression. Our findings demonstrate that acyl-CoA ligands can modulate FadR-DNA binding upstream from the *LEE1* promoter. We observed incomplete shifts in our EMSAs with the *LEE1* and *fadL* probes, which could be explained by the incomplete saturation of the FadR protein with the acyl-CoA ligands. Moreover, we have previously reported the presence of a second putative FadR binding site upstream from the *LEE1* promoter (15), which could also contribute to these incomplete shifts. Nonetheless, we also confirm long-chain acyl-CoA modulation of FadR-DNA binding at the *LEE1* promoter through CHiP quantitative real-time PCR (qRT-PCR) and thermal unfolding experiments. Our findings further demonstrate that in the presence of exogenous LCFAs, LEE expression is downregulated and follows the expression patterns of *fabB*, which is known to be positively regulated by FadR. Given that FadR binds its DNA targets in its apo form, our findings are consistent with the idea that FadR is an activator of the LEE. Notably, our previous work also demonstrated that genetic ablation of *fadR* in C. rodentium resulted in decreased activation of the LEE in the gut, which corresponded with attenuated disease (15). Finally, our previous findings further identified complex molecular cross talk between cysteine sensing, *fadL* expression, and FadR transcriptional modulation of the LEE in EHEC and C. rodentium (15). Taken together, our collective work strongly suggests that FadR is a pivotal regulator of the LEE that couples the direct and indirect sensing of various environmental signals to virulence.

Finally, our work demonstrates that the canonical LCFA machinery in EHEC can process and recognize arachidonic acid in addition to shorter-chain LCFAs such as oleic acid and palmitic acid. To our knowledge, sensing of arachidonic acid via direct recognition by the Fad or Fab enzymatic machinery has not been previously described in E. coli. Our genetic, biochemical, and functional studies together suggest that arachidonic acid is transported into the cell via the outer membrane protein FadL and then activated by the acyl-CoA synthetase FadD, which enables direct recognition by long-chain acyl-CoA sensor FadR. Structural and ligand binding studies of FadL suggest that its extracellular binding domain that initially interacts with exogenous LCFAs has a relatively low binding affinity for its substrates compared to other outer membrane transporters, suggesting that FadL may be capable of importing a wide range of hydrophobic substrates (18, 19, 63). Given that FadL can bind the 18-carbon-long UFA oleic acid (63), it is plausible that FadL also recognizes the 20-carbon-long PUFA arachidonic acid as suggested by our functional studies with the *fadL* mutant in EHEC. Structural and functional studies investigating E. coli FadR interactions with long-chain acyl-CoAs have demonstrated that the C terminus domain contains a binding pocket that interacts with the acyl-CoA moiety, which results in a conformational change that decreases FadR binding affinity to its target DNA motifs (25, 26, 64). Our work confirms that in addition to shorter-chain acyl-CoAs, FadR can also bind to AA-CoA, which alters its regulatory functions as a transcription factor. To our knowledge, the ability of E. coli FadD to utilize arachidonic acid as a substrate for acyl-CoA synthetase activity has not been previously reported. Components of the LCFA sensing and metabolic machinery are conserved among many *Enterobacteriaceae* pathogens and commensals, with homologs present in diverse bacterial species. Thus, in addition to its established effects on the host, arachidonic acid may modulate other aspects of bacterial function and physiology through FadR-dependent mechanisms in commensals and pathogens, further expanding its role as an important mediator in human health and disease.

## MATERIALS AND METHODS

### Bacterial strains, plasmids, and growth conditions.

The bacterial strains and plasmids used in this study are listed in Table S1 in the supplemental material. EHEC O157:H7 strain 86-24 and its isogenic mutants were routinely grown overnight in Luria broth at 37°C, with shaking at 250 rpm. For all experiments, unless otherwise indicated, EHEC strains were subcultured into Dulbecco modified Eagle medium (DMEM) with 1-g/liter glucose (Gibco) at 37°C for LEE-inducing conditions and incubated either as standing cultures (microaerobic) or with vigorous shaking at 250 rpm (aerobic). Unless otherwise indicated, EHEC was grown in the presence of 8 µM arachidonic acid (Sigma), 8 µM palmitic acid (Sigma), or the vehicle control (methanol, final concentration of 1:10,000).

10.1128/mBio.03247-20.5 TABLE S1 Bacterial strains used in this study. Download Table S1, DOCX file, 0.06 MB. Copyright © 2021 Ellermann et al. 2021 Ellermann et al. This content is distributed under the terms of the Creative Commons Attribution 4.0 International license.

### Construction of deletion mutants.

Isogenic mutants were generated using the lambda red recombinase method as described previously (65). Primers used to generate the linear DNA products and to genotype isogenic mutants are listed in Table S2.

10.1128/mBio.03247-20.6 TABLE S2 Oligonucleotide primers used in this study. Download Table S2, DOCX file, 0.06 MB. Copyright © 2021 Ellermann et al. 2021 Ellermann et al. This content is distributed under the terms of the Creative Commons Attribution 4.0 International license.

### RNA isolation and quantitative real-time PCR.

RNA was isolated using the RiboPure bacterial isolation kit (Ambion) per the manufacturer’s instructions. cDNA was synthesized using SuperScript II reverse transcriptase (ThermoFisher Scientific). qRT-PCR was performed in a QuantStudio 6 Flex instrument (Life Technologies) with Power SYBR green (Applied Biosystems) as follows: a single hold at 50°C for 2 min and at 95°C for 10 min, followed by 40 cycles at 95°C for 15 s and 60°C for 1 min. Each PCR was performed in 10-µl reaction mixtures and contained 1× SYBR green mix and 0.25 µM (each) primer. Melting curves were assessed to ensure specificity of the PCR products. Table S2 lists qRT-PCR primers used to amplify mRNA transcripts. The relative abundance of mRNA transcripts was calculated using the threshold cycle (ΔΔ*C_T_*) method and normalized to *rpoA* levels.

### Western blot assays for lysate-associated and secreted proteins.

Secreted proteins were isolated as previously described (49). Bovine serum albumin (BSA) was used as a loading control and added to secreted protein samples. Cell pellets were resuspended in 8 M urea to harvest lysate-associated proteins. Proteins were separated by a 4 to 15% gradient SDS-PAGE gel, transferred to a polyvinylidene fluoride membrane, and blocked with 5% milk or 5% BSA (as appropriate) in phosphate-buffered saline (PBS) with 0.05% Tween. Membranes were probed with anti-EspA, anti-EspB, or anti-Tir primary antibodies, followed by incubation with secondary antibodies conjugated to streptavidin-horseradish peroxidase. Membranes were exposed with the Bio-Rad ChemiDoc Touch imaging system with Image Lab 5.2.1 software for image analysis.

### FadR protein purification.

The FadR protein from EHEC 86-24 was purified as previously described. Briefly, to generate the N-terminal His-tagged FadR construct, the *fadR* gene from 86-24 was cloned into the NdeI and BamHI restriction enzyme sites in the plasmid pET28 by Gibson assembly per the manufacturer’s instructions (NEB). The pET28-*fadR* plasmid was then transformed into NiCO21 (NEB) chemically competent cells. Protein expression was performed as follows. Cultures were grown in Luria broth with 50 µg/ml kanamycin at 37°C with vigorous shaking until reaching an optical density at 600 nm (OD_600_) of about 0.6. The culture temperature was then dropped to 20°C, and a 1 mM concentration of the inducer isopropyl-β-d-thiogalactoside (IPTG) was added. Vigorous shaking was continued for 18 h. Cells were pelleted, washed with PBS, and resuspended in lysis buffer (20 mM Na_2_HPO_4_-KH_2_PO_4_ buffer at pH 7.7, 1.5 mM dithiothreitol [DTT], 20 mM imidazole) with protease inhibitor cocktail (Sigma). The cell suspension was lysed on ice by sonication on a Qsonica Q125 sonicator at 75% power for 6 min with 30-s on and 55-s off pulses. Lysates were cleared by centrifugation and filter sterilized. Samples were incubated with nickel-nitrilotriacetic acid (Ni-NTA) beads for 1 h at 4°C with shaking. Samples were applied to a gravity column and washed extensively with lysis buffer. Samples were eluted from the column with lysis buffer containing 250 mM imidazole. Samples were dialyzed and concentrated using 10,000-molecular-weight-cutoff (MWCO) Amicon spin concentrators into 20 mM Na_2_HPO_4_-KH_2_PO_4_ buffer at pH 7.7 and 1.5 mM DTT.

### Electrophoretic mobility shift assay (EMSA).

EMSAs were performed as previously described. DNA probes were prepared by PCR from 86-24 genomic templates as previously described. Briefly, DNA probes were purified by gel electrophoresis and labeled with l-[^32^P]ATP by T4 polynucleotide kinase (PNK) (NEB). Labeled probes were further purified using the Qiagen PCR purification kit per the manufacturer’s instructions. For EMSAs, labeled probe DNA was incubated at room temperature for 60 min with various concentrations of recombinant FadR protein in reaction buffer [50 mM Na_2_HPO_4_-KH_2_PO_4_ buffer at pH 7.7, 100 mM NaCl, 1.5 mM DTT, 100 µg/ml BSA, 250 µg/ml poly(dI-dC)]. Binding was resolved on 5% polyacrylamide gels in Tris-borate-EDTA (TBE). Gels were dried onto filter paper, exposed to phosphorimager screens, and assessed on the GE Amersham Typhoon 5 scanner.

### Thermal shift assay.

Thermal shift assays were performed as previously described (66). Purified FadR was used at a final concentration of 2 µM. SYPRO Orange (Invitrogen) was used at a final concentration of 20×. Experiments were carried out in 50 µl volumes in PBS and 1.5 mM DTT in 96-well optical reaction plates (Thermo Fisher Scientific). All samples were run in triplicate in the QuantStudio 6 Flex instrument (Applied Biosystems). Fluorescence intensity was measured via the JOE emission filter (550 nm) and “PTS clear plate” was set as the background for the calibration. Temperature was continuously increased at 0.5°C/minute throughout the incubation. The following ligands were added at a concentration of 20 µM: palmitic acid in MeOH (Sigma), arachidonic acid in MeOH (Sigma), palmitoyl-CoA in H_2_O and arachidonoyl-CoA in H_2_O (Sigma). Melting curves were directly exported from the instrument and were analyzed using Prism 6 (GraphPad Software Inc.).

### Isothermal titration (ITC) calorimetry.

Calorimetric measurements were carried out as previously described (54), using an SV-ITC microcalorimeter (MicroCal). The reference cell was filled with PBS. The calorimeter was electrically calibrated at each temperature. All solutions used for the experiments were thoroughly degassed by stirring under vacuum. If necessary, protein solutions were spun for several minutes in a benchtop centrifuge to remove any visible particles. The concentration of the protein was estimated spectrophotometrically at 280 nm using 33,060 M^−1^ cm^−1^ as the extinction coefficient for recombinant FadR. Purified FadR in PBS was placed in the sample cell. The ligands palmitoyl-CoA (Sigma) and arachidonoyl-CoA (Sigma) were dissolved in the same buffer as the protein and were drawn into the injection syringe, which was then mounted into a stepper motor for delivery into the sample cell. The syringe with stirrer paddle was rotated at 400 rpm during the experiment to ensure immediate mixing. Experiments were performed at 20°C. The concentration of the ligands, about 0.5 mM, was chosen to ensure full saturation well before final injection. Appropriate blank runs were conducted and subtracted from the corresponding data. All data were integrated using NITPIC (67) and analyzed in SEDPHAT (68).

### Acyl-CoA synthetase (ACS) activity measurement.

Measurement of ACS activity was performed as described previously (69). Briefly, lysates of WT 86-24 and Δ*fadD* 86-24 lysed with KTx buffer (130 mM KCl, 25 mM Tris-HCl at pH 7.4, 1% Triton X-100) were incubated for 10 min at 30°C with the following reaction mix: 100 mM Tris-HCl at pH 7.4, 5 mM MgCl_2_, 200 µM DTT, 10 mM ATP, 200 µM CoA, 0.1% Triton X-100, and 20 µM [^14^C]oleate (PerkinElmer) or [^14^C]arachidonic acid (PerkinElmer) bound to 5 mM fatty acid-free BSA. The reaction was terminated by addition of Dole’s solution (isopropanol:heptane:H_2_SO_4_, 40:10:1 [vol/vol]). Free fatty acids were extracted by five washes with heptane. The radioactivity of the aqueous phase, corresponding to the amount of synthesized oleoyl-CoA or arachidonoyl-CoA, was determined by liquid scintillation counting (LS 6500; Beckman-Coulter, Brea, CA).

### Chromatin immunoprecipitation (ChIP) qRT-PCR.

The pASKIBA32::FadR-V5 plasmid, encoding FadR with an N-terminal V5 tag, was transformed into the Δ*fadR* 86-24 strain for ChIP assays as previously described. Overnight cultures of 86-24 Δ*fadR* pASKIBA32::FadR:V5 were diluted 1:100 into 60 ml of low-glucose DMEM microaerobically as described above. After 4 h of growth with shaking, the protein-DNA complexes in the bacterial cells were cross-linked *in vivo* with 1% formaldehyde at room temperature for 20 min. Cross-linking was stopped by addition of 500 mM glycine. Bacteria were then washed twice with cold PBS and resuspended in 1 ml of IP buffer (10 mM Tris at pH 8, 150 mM NaCl, 1 mM EDTA, 1% Triton X-100, 0.1% deoxycholate, 0.1% RNase A, 1 µl/ml of protease inhibitor cocktail) and sonicated into fragments of 100 to 600 bp on a Qsonica Q125 sonicator using the following parameters: 7 cycles of 30 s on and 30 s off at 95% power. Insoluble cellular debris was removed by centrifugation, and the supernatant was used as the input sample in IP experiments. Dynabeads (1.5 mg protein A; Sigma) were loaded with 10 µg of anti-V5 antibody (Sigma) and 1 mg/ml poly(dI-dC). Then, IP samples were incubated overnight with the loaded Dynabeads. After incubation, the beads were washed twice with IP buffer followed by eight washes with the LiCl wash (10 mM Tris at pH 8, 500 mM LiCl, 1 mM EDTA, 0.5% Nonidet P-40, 0.5% sodium deoxycholate) and once with Tris-EDTA (TE) buffer. The beads were resuspended in 100 µl of elution buffer (10 mM Tris at pH 8, 50 mM NaCl, 10 mM EDTA, 1% SDS), incubated for 1 h at 65°C, and centrifuged at 5,000 × *g* for 1 min. The supernatants were incubated at 65°C overnight or boiled at 95°C for 10 min to reverse the DNA-protein cross-links. Then, 1 µl of RNase A was added, and the solutions were incubated for 30 min at 37°C. Four microliters of 0.5 M EDTA, 8 µl of 1 M Tris-HCl, and 1 µl of proteinase K were added to each tube and incubated at 45°C for 2 h. The DNA was purified using phenol-chloroform or Qiagen MinElute kits. We then proceeded with qRT-PCR used to evaluate the percentage of input of each sample captured during ChIP, using primers that span the FadR protein binding site in the *fadL* and *ler* promoters (Table S2).

### FAS assay.

Fluorescent actin staining (FAS) assays were performed on HeLa cells as described previously (4). Briefly, HeLa cells were grown to 80 to 90% confluence on coverslips in DMEM with 10% fetal bovine serum (FBS) and 1% penicillin-streptomycin. Three hours prior to infection, epithelial cells were incubated in DMEM with 0.1% glucose and without serum and antibiotics. Prior to infection, mCherry-expressing bacteria were subcultured in DMEM with 0.1% glucose and arachidonic acid or the vehicle control for 4 h. Epithelial cells were then infected at a multiplicity of infection (MOI) of 10. Following 4 h of infection, the samples were washed, fixed, permeabilized, and stained with fluorescein isothiocyanate (FITC)-labeled phalloidin and 4′,6-diamidino-2-phenylindole (DAPI). Images for pedestal enumeration were taken using the Zeiss LSM780 confocal/multiphoton microscope at the UT Southwestern Live Cell Imaging Core Facility. Pedestal formation was quantified in two ways—as the number of pedestals per infected cell and the percentage of epithelial cells that contain pedestals.

### Epithelial adhesion assay.

EHEC adhesion assays were performed on HeLa or Caco-2 cells as previously described (70). Briefly, epithelial cells were grown to 80 to 90% confluence in DMEM with 10% FBS and 1% penicillin-streptomycin. One hour prior to infection, epithelial cells were serum starved and incubated in the absence of antibiotics. Prior to infection, EHEC was subcultured in DMEM with 0.1% glucose and arachidonic acid or the vehicle control for 3 to 4 h. Epithelial cells were then infected at an MOI of 10 for 4 h, followed by PBS washes to remove loosely attached bacteria. Epithelial cells were then lysed with Triton X-100, and remaining epithelial cell-associated bacteria were enumerated by quantitative bacterial culture.

### Statistical analysis.

The statistical tests utilized are indicated in each figure legend. Generally, *P* values were calculated using Student’s *t* test when 2 experimental groups were compared and one-way analysis of variance (ANOVA) with Bonferroni multiple-comparison posttest when 3 or more experimental groups were compared. All enumeration of bacteria by serial dilution and plating was log transformed to normalize the data. For microscopy and densitometry analyses, *P* values were calculated using the Mann-Whitney test when 2 experimental groups were compared and the Kruskal-Wallis test with the Dunn posttest when 3 or more experimental groups were compared.

### Data and material availability.

All data, materials, and strains published in this article are available upon request.
