# Supplementary figures and images for: The Canonical Long-Chain Fatty Acid Sensing Machinery Processes Arachidonic Acid To Inhibit Virulence in Enterohemorrhagic Escherichia coli
Source: mBio. 2021 Jan 19;12(1):e03247-20. doi: 10.1128/mBio.03247-20 (PMC7845647; doi:10.1128/mBio.03247-20)

**A** Secretion profile

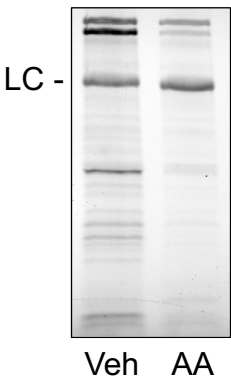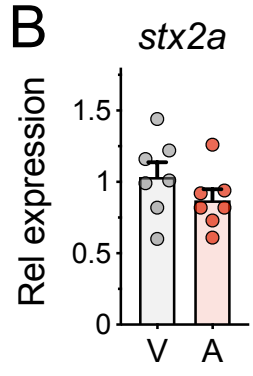

Supplement: FIG S1 [file mBio.03247-20-sf001.pdf]

A

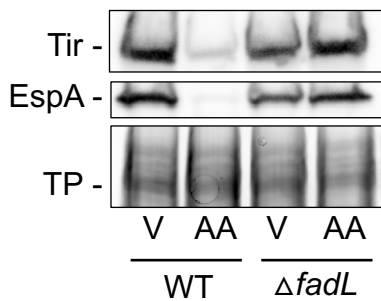

# B

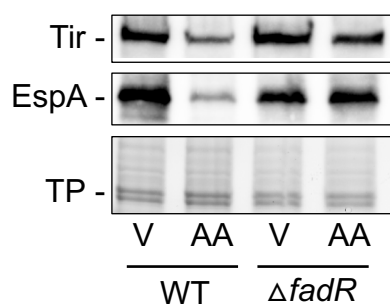

C

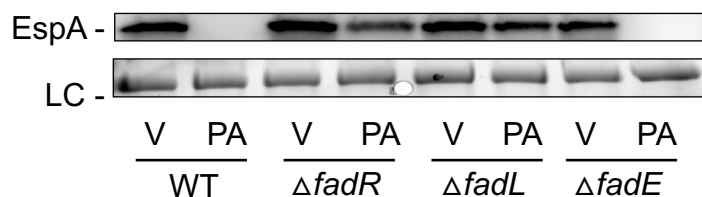

D

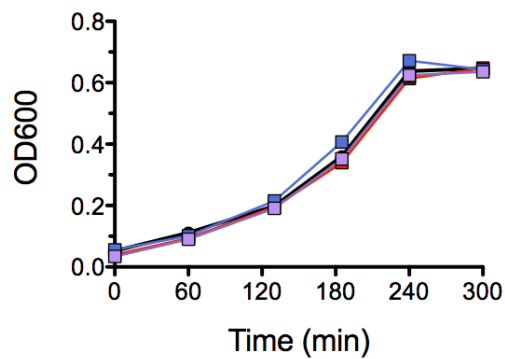

# E

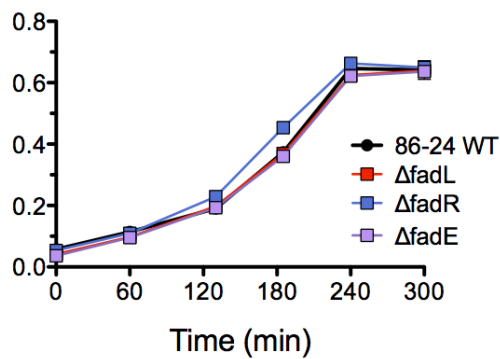

Supplement: FIG S2 [file mBio.03247-20-sf002.pdf]

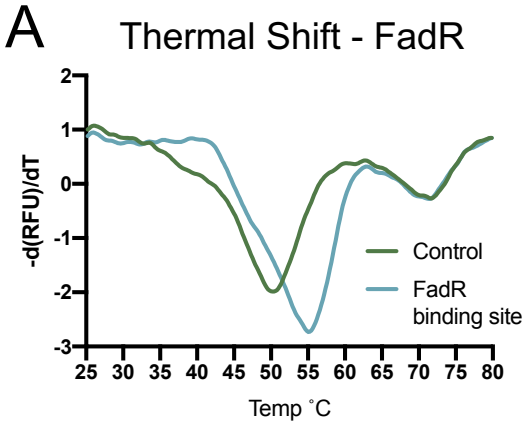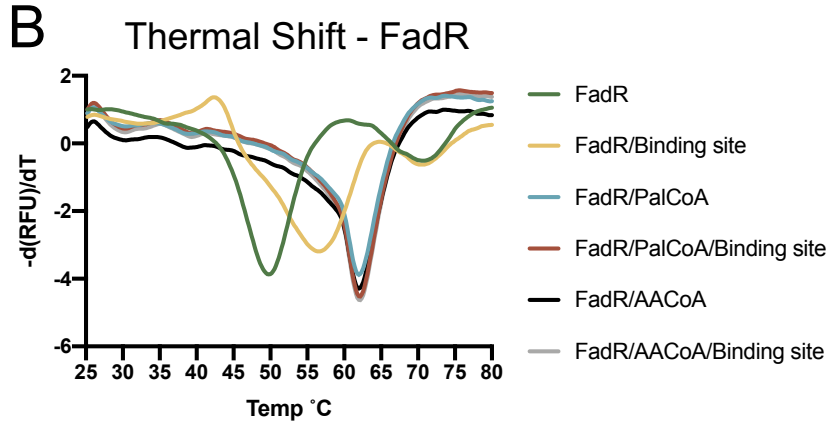

**C** EMSA – *kan* promoter

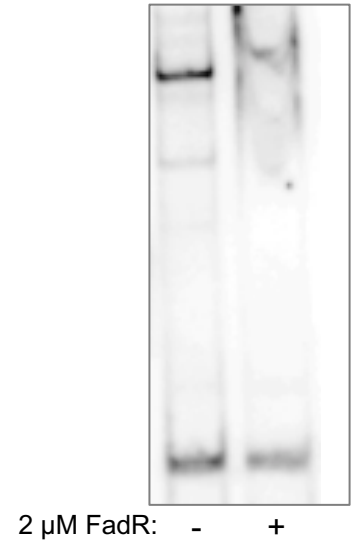

Supplement: FIG S3 [file mBio.03247-20-sf003.pdf]

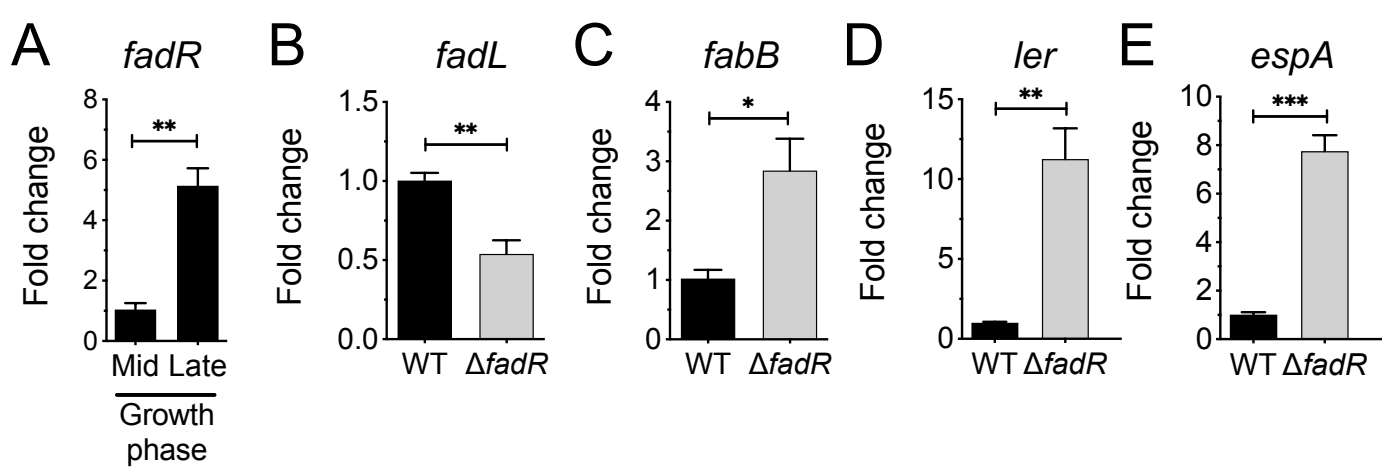

Supplement: FIG S4 [file mBio.03247-20-sf004.pdf]
