# Supplementary material for: The Canonical Long-Chain Fatty Acid Sensing Machinery Processes Arachidonic Acid To Inhibit Virulence in Enterohemorrhagic Escherichia coli
Source: mBio. 2021 Jan 19;12(1):e03247-20. doi: 10.1128/mBio.03247-20 (PMC7845647; doi:10.1128/mBio.03247-20)
Supplement: TABLE S1 [file mBio.03247-20-st001.docx]

**TABLE S1** Bacterial strains used in this study

| **Strains** | **Description** | **Reference** |
| --- | --- | --- |
| *E. coli* 86-24 | Shigatoxin-positive EHEC strain serotype O157:H7, streptomycin resistant | (1) |
| 86-24 *fadR* | *fadR* isogenic mutant in 86-24 | (2) |
| 86-24 *fadL* | *fadL* isogenic mutant in 86-24 | (2) |
| 86-24 *fadE::kan* | *fadE* isogenic unresolved mutant in 86-24 | This study |
| 86-24 *escN* | *escN* isogenic mutant in 86-24 | This study |
| 86-24 *espA* | *espA* isogenic mutant in 86-24 | (3) |
| 86-24 *fadD::kan* | *fadD* isogenic unresolved mutant in 86-24 | This study |
| mCherry-expressing  86-24 | 86-24 transformed with pDP151 | (4) |
| 86-24 *fadR* FadR::V5 | *fadR* isogenic mutant in 86-24 transformed with FadR::V5 expression vector pRP007 | (2) |
| NiCo21(DE3) pET28 FadR | FadR protein expression strain | (2) |
| *S. aureus* |  | Kind gift from Dr. Julie Pfeiffer |

**References**

1. Griffin PM, Ostroff SM, Tauxe RV, Greene KD, Wells JG, Lewis JH, Blake PA. 1988. Illnesses associated with Escherichia coli O157:H7 infections. A broad clinical spectrum. Ann Intern Med 109:705–12.

2. Pifer R, Russell RM, Kumar A, Curtis MM, Sperandio V. 2018. Redox, amino acid, and fatty acid metabolism intersect with bacterial virulence in the gut. Proc National Acad Sci 115:201813451.

3. Cameron EA, Curtis MM, Kumar A, Dunny GM, Sperandio V. 2018. Microbiota and Pathogen Proteases Modulate Type III Secretion Activity in Enterohemorrhagic Escherichia coli. Mbio 9:e02204-18.

4. Ellermann M, Pacheco AR, Jimenez AG, Russell RM, Cuesta S, Kumar A, Zhu W, Vale G, Martin SA, Raj P, McDonald JG, Winter SE, Sperandio V. 2020. Endocannabinoids Inhibit the Induction of Virulence in Enteric Pathogens. Cell https://doi.org/10.1016/j.cell.2020.09.022.
