# Supplementary material for: The Canonical Long-Chain Fatty Acid Sensing Machinery Processes Arachidonic Acid To Inhibit Virulence in Enterohemorrhagic Escherichia coli
Source: mBio. 2021 Jan 19;12(1):e03247-20. doi: 10.1128/mBio.03247-20 (PMC7845647; doi:10.1128/mBio.03247-20)
Supplement: TABLE S2 [file mBio.03247-20-st002.docx]

**TABLE S2** Oligonucleotide primers used in this study

| **Name** | **Sequence (5’ to 3’)** | **Reference** |
| --- | --- | --- |
| EHEC *espA* | FP – AGCTATTTGAGGAACTCGGTG  RP – CATCTTTTGTGCCGTGGTTG | (1) |
| EHEC *tir* | FP – GAGGGAGTCAAATAGCGGTG  RP – ATCTGAACGAAGGCTGGAAG | (1) |
| EHEC *escC* | FP – CTGAAGACAATGGCAAGTAATGG  RP – ACTGCATTAAGACGTGGATCAG | (1) |
| EHEC *escV* | FP – GAGTGCAAAAGGAAAGCCAG  RP – ATGATACCAGCAATAGCGTCC | (1) |
| EHEC *rpoA* | FP – GTGACCCTTGAGCCTTTAGAG  RP – ACACCATCAATCTCAACCTCG | (1) |
| EHEC *fadL* | FP – CGTCTCCATCTGGTCGTAGC  RP – AGTGCATGTTCGGAACCCAT | This work |
| EHEC *fabB* | FP – atgccatcgctttggtgacc  RP – cgtgttggcctgattgcagg | This work |
| FadR_*fadL* site | FP – ATCTGGTACGACCAGAT  RP – ATCTGGTCGTACCAGAT | This work |
| FadR_*ler* site | FP – TAATCGTTTGTCCGACTTTAACAGA  RP –TCTGTTAAAGTCGGACAAACGATTA | This work |

**References**

1. Jimenez AG, Ellermann M, Abbott W, Sperandio V. 2019. Diet-derived galacturonic acid regulates virulence and intestinal colonization in enterohaemorrhagic Escherichia coli and Citrobacter rodentium. Nat Microbiol 5:368–378.
